# Medical Opinion and Movement

**Published:** 1906-11-10

**Authors:** 


### Medical Opinion and Movement.

The decision of the Representative meeting of the British Medical Association in July last, that the Association should take up a neutral position in regard to the election of direct representatives to the Medical Council, has not been adhered to by some branches. At Swansea and at Wandsworth and elsewhere the local branches have approved certain selected candidates, and announced that approval to the profession through the press and in other ways. This course, we think, must tend to diminish the legitimate influence of the British Medical Association, and we regret it accordingly. We cannot, however, follow the argument that, when a practitioner is a member of the staff of a great provident dispensary, and receives some hundreds a year for the medical services he renders, he is ineligible to stand as a direct representative, because he has been guilty of unfair competition with outside practitioners in the locality where such provident dispensary is situated. We believe, for instance, that the Battersea Provident Dispensary, with a membership of over 20,000, is well managed, and that in many ways it has rendered good service to the profession and the public.

Dr. William R. Bennett calls attention to a matter which affects the interests of medical practitioners. One reason why the income of general practitioners steadily decreases is, in Dr. Bennett's opinion, the reckless ordering of "tabloid" preparations, for which the practitioner himself is wholly responsible. The medical man orders these tabloids at the outset either verbally or by prescription, and the patient obtains them often in the original bottles, the word "tabloid" being stamped upon them, and he so learns that they are readily obtainable from any chemist by "every man, woman, or child" who produces the money to pay for them. The ordering of tabloids not only damages each medical man's own practice, but gives them an excellent advertisement, and he so acts "as a first-rate traveller for the wholesale firm whose goods he so constantly pushes." Dr. Bennett urges practitioners to prescribe all compressed drugs as *tabellæ*, and to send the patient to a reliable pharmacist. "Medical practitioners being entirely responsible for the introduction of tabloids should, by concerted action, now become responsible for their disappearance."

Sir Dyce Duckworth has done good service in the address he delivered in Edinburgh to the Royal Medical Society by raising the question, Is medicine regulated nowadays with the respect due to it? What the medical profession needs at the moment is a statesman or statesmen within its ranks, who will sacrifice their comfort to the well-being of the whole profession, by persistently leading the public to a higher appreciation of the importance of authority in medicine. They might so cause all classes to awaken to a realisation of the fact, that self-medication is harmful in itself, and in these days probably leads to the production of more unhealthiness and bodily discomfort, than any other habit acquired by the human race. Elegant preparations, though they have done much to make the administration of remedies easy, have also contributed to increase the habit of taking drugs without medical control, to an extent which causes many people to become mere physical wrecks, rather than healthy men and women. Sir Dyce Duckworth's warning note should be taken up by other leaders of medical opinion and by every reputable newspaper. The more it is driven home the better will it be for the nation and for all classes of the people.

Dr. William Bruce sends a most instructive letter on the pathology of sciatica to last week's *Lancet*. Dr. Bruce's view is that very many cases of sciatica are due to trouble in the hip joint, and that in all such cases each patient should be examined systematically in order to discover whether the hip joint on the particular side affected is diseased. The danger in these cases is to over-generalise, and to found the diagnosis and treatment on settled opinions, while too seldom care is taken to study, thoroughly, point by point, each individual case. Dr. Bruce records a case of an elderly man, who had been ailing for fully four years, beginning with pains in the back, shooting down his thighs. Under advice he had tried baths, taken waters and been systematically douched, with no improvement. On examination Dr. Bruce found double rheumatoid arthritis of the hip joint and consequent ankylosis, as the result of the absence of adequate treatment, during the years in question. Dr. Bruce maintains, as the result of his experience, that with proper steady massage the disease might have been, if not cured, at least stayed in its fatal progress. He further insists, that parallel mistakes may occur in so-called neuritis of the arm, and insists, that in every such case, diligent inquiry and examination should be made as to the physical condition of the affected limb, and more particularly of the shoulder joint. Unless the patient complains of pain in the latter, gouty arthritis of the shoulder is exceedingly apt, in Dr. Bruce's opinion, to be missed. Placing one hand on the scapula and keeping that bone in its place, elevation of the limb affected, at once reveals, in many of these cases of neuritis, more or less (false) ankylosis of the shoulder joint. Tenderness may often be discovered on pressure over the front and thinner portion of the capsule. These symptoms are often quite decided signs of disease of the nature indicated. He quotes another case, that of a lady with these conditions, who consulted him years ago, who could not put her dress on or off by herself. Though she is now 80 years of age her shoulder joint is all right, and this result is undoubtedly due, mainly, to skilled massage, aided by the internal use of the sulphur waters, and the outward application of baths and warm douches. Dr. Bruce's great experience, and the practical nature of his communication, have led us to call attention to the advice he gives, in regard to a class of cases, which are bound to go wrong, if a careful diagnosis is not formed, at the commencement of treatment.

